# In situ analysis of FOXP3+ regulatory T cells in human colorectal cancer

**DOI:** 10.1186/1479-5876-4-52

**Published:** 2006-12-13

**Authors:** Christoph Loddenkemper, Martin Schernus, Michel Noutsias, Harald Stein, Eckhard Thiel, Dirk Nagorsen

**Affiliations:** 1 Department of Pathology, Charité -Universitätsmedizin Berlin, Campus Benjamin Franklin, Berlin, Germany; 2 Department of Hematology, Oncology, and Transfusion Medicine, Charité -Universitätsmedizin Berlin, Campus Benjamin Franklin, Berlin, Germany; 3 Department of Cardiology and Pneumonology, Charité -Universitätsmedizin Berlin, Campus Benjamin Franklin, Berlin, Germany

## Abstract

The immune system spontaneously responds to tumor-associated antigens in peripheral blood of colorectal cancer (CRC) patients. Regulatory T cells (Treg) are suspected of influencing the interaction between the tumor and immune system and thus the course of malignant diseases. However, the function of Tregs in the development of T cell responses and on the clinical course of CRC is not clear. We analyzed Treg infiltration (FOXP3 staining) *in situ *in 40 CRC patients and investigated whether there is a correlation to disease stage, systemic T cell response, and survival. Treg infiltration was significantly higher in CRC than in healthy colon. Stromal Treg infiltration was significantly higher than epithelial infiltration in CRC. Furthermore, Treg infiltration in the tumor was significantly higher in limited disease than in metastatic CRC. The average Treg infiltration rate in the tumor was non-significantly higher in patients without systemic TAA-specific T cell response. Survival did not differ between patients with high Treg infiltration and those with low Treg infiltration. In conclusion, a direct link between Treg infiltration in the tumor and the development of a systemic T cell response in CRC cannot be proven. However, local Treg infiltration was significantly higher in limited disease, in which a systemic TAA-directed T cell responses is less frequently observed.

## Background

In the last years, immunotherapy of malignant diseases and the understanding of underlying mechanisms have made rapid progress despite limited clinical response [1]. T cells directed against tumor antigens are believed to play a crucial role in mediating anti-tumor effects. Even without prior immunotherapy, spontaneous T cell responses against tumor antigens have been described in a variety of malignant diseases [2]. Colorectal cancer (CRC) has been intensely studied for interactions with the immune system. Although the colon is considered a rather tolerogenic organ, spontaneous T cell responses against several tumor-associated antigens (TAA) have been detected in the peripheral blood of CRC patients, particularly in metastatic disease [3,4]. However, patients with peripheral TAA-directed T cell response showed no benefit in a first survival analysis with a limited number of CRC patients [5]. Interestingly, active specific immunotherapy of metastatic CRC only causes a clinical response in less than 1% of patients despite the successful induction of immunological responses [6]. Multiple factors like the immune system, tumor stroma, and tumor cells influence the induction and modulation of tumor-directed immune responses [7]. Limited anti-tumor activity of antigen-specific T cells at a clinical level may be limited in CRC patients for various reasons. One major reason for the clinical failure of tumor-directed immune responses might be the immunosuppressive effect of tumor-infiltrating regulatory T cells.

The immune system is potentially able to develop immune responses against self antigens and tumor-associated antigens. Regulatory T cells (Tregs) play a crucial role in regulating the balance between the attack and tolerance of such antigens. Regulatory CD4+ CD25+ FOXP3+ T cells are a subset of T cells mediating immunological tolerance by suppressing the activation of autoreactive T cells. Tregs not only inhibit the development of autoimmune-mediated diseases such as inflammatory bowel disease or intestinal graft-versus-host disease [8,9] but are also suspected of impeding immunosurveillance against autologous tumor cells [10]. Tregs have been found to be increased in the peripheral blood of patients suffering from various malignant diseases. Wolf et al. reported an increase of Tregs in peripheral blood of patients with epithelial malignancies [11]. Sasada et al. [12] described an increased percentage of Tregs among lymphocytes in peripheral blood of patients with gastrointestinal cancers. An increase of Tregs in peripheral blood has also been reported for hepatocellular cancer and non-small cell lung cancer [13,14]. Kono et al. [15] found higher Tregs in patients with advanced gastric and esophageal cancer than in those with limited disease. Ichihara et al. [16] reported an increase of Tregs not only in the peripheral blood of patients with gastric and esophageal cancer but also in the tumor itself compared to healthy mucosa. Moreover, they demonstrated a higher Treg infiltration in advanced gastric/esophageal cancer [16]. All of this suggests a role of Tregs in tumor development, which may be related to immunosuppression in humans.

Several mouse studies have shown that depletion of regulatory T cells improves anti-tumor immunity [17-19]. For example, Ko et al. [20] found FOXP3+CD25+CD4+ Tregs predominantly in growing tumors in mice that had not received an agonistic monoclonal antibody against glucocorticoid-induced tumor necrosis factor receptor family-related protein (GITR), thus suggesting that tumor-infiltrating natural Tregs may hamper effective tumor immunity. A similar conclusion can be drawn from the work of Turk et al. [21], which shows that melanomas were immunologically rejected after Treg inhibition, and an improvement of anti-tumor T cell response was obtained when Tregs had been depleted before immunization of mice. In a murine colon cancer model, Ghiringelli et al. [22] demonstrated that Tregs suppress anti-tumor immunity and that this effect can be circumvented when Tregs are depleted by cyclophosphamide. This stimulated intensified research on the interaction between Tregs and adoptive CD8+ T cell transfer. Antony et al. [23] described a reduced effect of adoptively transferred tumor-antigen-directed CD8+ T cells when given together with Tregs in mice. Interestingly the *in vitro *induction of tumor-antigen-specific (anti-NY-ESO-1) antibodies required Treg depletion from seronegative cancer patients and healthy donors [24]. This is in accordance with data by Rosenberg & Dudley [25], which suggest that Treg depletion may also be a prerequisite for a clinically effective adoptive T cell transfer in humans.

As mentioned above, Tregs are suspected of reducing T cell activity, but their role in the development of tumor-antigen-specific T cell responses has not yet been investigated in detail. Furthermore, it is not known whether the intratumoral presence of Tregs has an impact on the antigen-specific T cell response, clinical course, and survival of CRC patients. With regard to tumors of other organs, Albers et al. [26] described an increased number of regulatory and antigen-specific T cells in head and neck cancer. Yu et al. [27] observed Treg accumulation at the tumor site. They were able to show in a murine fibrosarcoma model that Treg inhibition resulted in a changed cytokine milieu in the tumor and an eradication of tumors. Viguier et al. (28) reported that the number of FOXP3 expressing regulatory T cells is increased in human metastatic melanoma lymph nodes. These cells inhibited *in vitro *proliferation and cytokine production of CD8 +T cells [28]. It was shown for ovarian cancer that tumoral Tregs suppress tumor-specific T cell immunity and contribute to reduced survival [29]. Another study on ovarian cancer patients found that not only intraepithelial CD8+ tumor-infiltrating lymphocytes but also a high CD8+/CD25+FOXP3+ regulatory T cell ratio (i.e., low relative FOXP3 infiltration) is associated with a favorable prognosis [30]. Moreover, Wolf et al. [31] reported that a high expression level of FOXP3 in ovarian cancer is associated with a poorer prognosis. Briefly, the latter three studies suggest that high Treg infiltration is associated with a poorer prognosis in ovarian cancer. Overall, targeting Tregs may have an important impact on immunotherapeutic anti-cancer strategies and the clinical outcome of cancer patients [19].

The aim of our study was to analyze the infiltration of CRC by FOXP3-positive Tregs *in situ *and to investigate whether there is a correlation to disease stage, systemic TAA-specific T cell response, and survival of CRC patients. We applied an immunohistochemical double labeling method using anti-FOXP3-Ab and anti-CD3-Ab; and since CD8+ T cell infiltration has been described as a positive prognostic factor in CRC [32-37], we additionally examined CD8 T cell infiltration in relation to Treg frequency, disease stage and survival.

## Materials and methods

### Patient selection and T cell assays

After receiving institutional review board approval and informed consent, we collected and froze peripheral blood mononuclear cells from CRC patients for T cell analysis. All analyses were performed in compliance with the Helsinki Declaration. HLA-A2-positive patients had been tested for the presence of T cell responses against the HLA-A*0201 presented T cell epitopes Ep-CAM p263-271, her-2/*neu *p654-662, and CEA p571-579 by ELISPOT assay. These T cell responses have been previously reported in more detail; and HLA analysis and ELISPOT were performed as previously described [3,4]. Positive T cell responses were also defined as described elsewhere [3-5]. Inclusion criteria were sufficient tumor sections for immunohistochemistry and sufficient clinical data. As a control group, we have analyzed biopsies from 12 healthy donors who underwent colonoscopy for CRC screening.

### Immunohistochemistry

Immunostaining was performed according to a previously published protocol with slight modifications [38]. Briefly, 4 μm-thick serial sections were cut, deparaffinized and subjected to a heat-induced epitope retrieval step before incubation with antibodies. Sections were immersed in sodium citrate buffer solutions at pH 6.0 and heated in a high-pressure cooker. The slides were incubated with the monoclonal antibody against CD8 purchased from Dako, Glostrup, Denmark (clone C8/144B, dilution 1:100). The alkaline phosphatase anti-alkaline phosphatase complex (APAAP) method was used for detection. Alkaline phosphatase was revealed by Fast Red as chromogen. For double immunoenzymatic labeling of FOXP3/CD3, slides were blocked using a peroxidase blocking reagent (Dako) and incubated for 30 minutes with the rat monoclonal antibody against the amino terminus of the human FOXP3 protein (PCH101, 1:500; eBioscience, San Diego, USA), followed by biotin-conjugated rabbit anti-rat (Dako, 1:200) and the EnVision peroxidase kit (Dako). Sections were then incubated for 30 minutes with the second antibody against CD3 (clone UCHT1, 1:25, Dako) and visualized by the APAAP method. Tonsillar tissue with follicular hyperplasia served as a positive control displaying scattered T cells in the interfollicular area with nuclear expression of FOXP3. Negative controls were performed by omitting primary antibodies. Ten randomly selected high power fields (1 HPF = 0,237 mm^2^) were analyzed for T cell infiltration in both the epithelium and stroma/lamina propria, and 10 HPF were averaged in each case.

### Statistical and survival analysis

#### Treg infiltration and systemic T cell response

Tregs were either analyzed separately as epithelial or stromal infiltration or as the sum of both as total Treg infiltration. The local Treg infiltration of patients with and without CRC was compared using a two-sided Student's t-test. Epithelial and stromal Treg infiltration in CRC was also compared using a two-sided Student's t-test. For the respective further analyses, CRC patients were grouped according to their positive or negative peripheral T cell response.

#### T cell (Treg, CD3, CD8) infiltration and survival

All patients were grouped according to their T cell infiltration. The median was used to separate patients with high infiltration from those with low infiltration. A Kaplan-Meier analysis was performed to compare survival of patients with high and low infiltration. Statistical significance was determined by the log rank test. All calculations for CD8 infiltration are descriptive post-hoc analyses, since Treg infiltration was the primary endpoint of our study. The association between Treg/CD8 ratio and survival was calculated in this way.

#### Stage correction

To reduce the influence of disease stage as prognostic factors, we grouped patients according to their disease stage. Stage correction was performed by categorizing patients according to their UICC stage. UICC I and II were combined because only one patient was UICC I. These stage-grouped patients were sorted according to their respective T cell infiltration. The median was used again to separate patients into low and high infiltration groups. All patients with low infiltration of stage group I+II, III, and IV were combined to make a new "stage-corrected low infiltration group". The same was done for a "stage-corrected high infiltration group". These two groups were then compared using Kaplan-Meier survival analysis. Statistical significance was determined by the log rank test.

#### Association between T cell infiltration, stage, age and gender and the correlation between Treg and CD8 infiltration

To analyze differences between T cell infiltration and stage, patients were grouped according to limited (UICC I and II) and metastatic (UICC III and IV) disease. These two groups were compared for tumoral and stromal infiltration by Tregs and CD8 T cells using the Student's t-test. A correlation (Pearson) was calculated for Treg and CD8 infiltration for both stromal and epithelial tumor infiltration. Moreover, we tested the association between T cell infiltration and age (Pearson correlation) and gender (t-test).

P < 0.05 was considered significant and p ≤ 0.1 a trend. SPSS software was used. All analyses other than Treg infiltration/disease stage, Treg infiltration/systemic T cell response and Treg infiltration/survival are descriptive because they represent post hoc analyses.

## Results

### Patient characteristics

Forty CRC patients met all inclusion criteria. One patient had UICC I, 20 patients UICC II, six patients UICC III, and 13 patients UICC IV. Eleven patients tested positively for a systemic TAA-specific T cell response to HLA-A2-binding peptides of CEA, Ep-CAM, and/or her-2/neu. Twenty-one patients were female and nineteen male. Mean age at first diagnosis was 62.1 years (patients with T cell response: 61.7 years, patients without T cell response: 62.2 years). As a control, we analyzed the colon biopsies of twelve healthy donors who underwent screening colonoscopy without evidence for CRC. Mean age of these donors was 64.6 years, (7 males, 5 females). See also table 1.

**Table 1:** FOXP3 infiltration

	**n**	**FOXP3/HPF ± SD**	**P value**
**Healthy controls**	12	0.6 ± 0.3	
Mean age 64.6 years, 5 female, 7 male			
			< 0.01
**CRC**	40	12.9 ± 9.1	
Mean age 62.1 years, 21 female, 19 male			
Stromal	40	10.8 ± 8.4	< 0.01
Epithelial	40	2.0 ± 2.2	
Limited (UICC I+II)	21	17.8 ± 10.6	0.04
Metastatic (UICC III+IV)	19	9.9 ± 6.1	
Positive T cell response	11	9.6 ± 7.3	0.16
Negative T cell response	29	14.1 ± 9.5	

### Treg infiltration in CRC and normal colon

Treg infiltration was found in all 40 CRC samples. The mean total Treg infiltration in CRC was 12.9/HPF while in healthy colon tissue a mean total Treg infiltration of 0.6/HPF was found. This difference was highly significant (p < 0.01). See also table 1.

### Treg infiltration in stroma and epithelium

**Figure 1:**
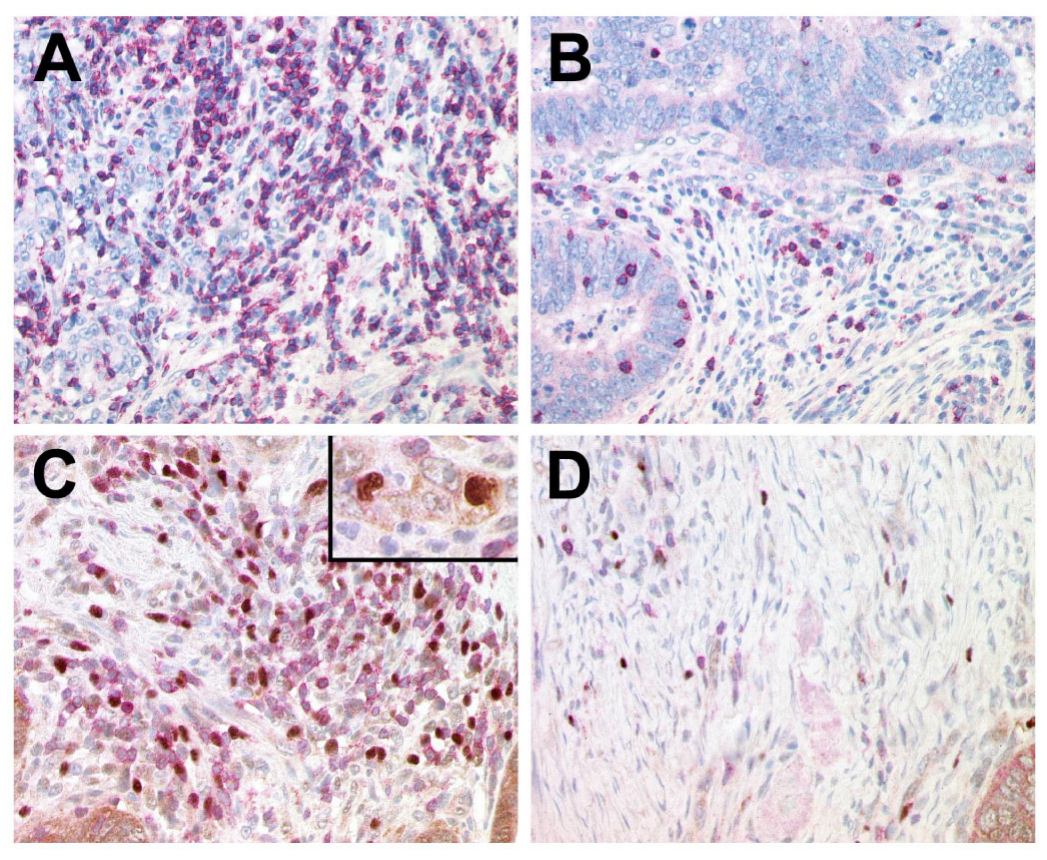
Immunohistochemical labeling of CD8+ and CD3+/FOXP3+ T cells of representative colon cancer specimens with one case demonstrating a high number of CD8+ T cells (A) and CD3+ T cells (red, membranous) with a high proportion of regulatory T cells co-expressing FOXP3 (brown, nuclear) in the stroma (C) and the malignant epithelium of the tumor (inset) and another case with a low number of CD8+ T cells (B) and only a few CD3+ T cells and FOXP3+ Treg (D).

The stromal Treg infiltration (mean 10.8/HPF) was significantly higher than the epithelial Treg infiltration (2.0/HPF) in CRC (p < 0.01). In healthy controls, we found a mean of 0.1 Tregs/HPF in the epithelium and 0.6 Tregs/HPF in the lamina propria of healthy donors. For both the epithelium and stroma/lamina propria, Treg infiltration was significantly higher (p < 0.01) in the tumor than in healthy colon. Representative samples are given in figure 1.

### Treg infiltration and CRC stage

Total Treg infiltration was significantly higher in limited disease than in metastatic CRC (17.8/HPF vs. 9.9/HPF, p = 0.04). This difference was mainly based upon infiltration pattern in stroma (15.8/HPF vs. 7.8/HPF; p = 0.03).

### Treg infiltration and systemic T cell response in CRC

The mean total Treg infiltration in patients without TAA-specific T cell response was 14.1/HPF, in patients with a TAA-specific T cell response 9.6/HPF. This difference, however, did not reach significant level (p = 0.16). In both tumor epithelium and stroma, the average Treg infiltration rate was slightly higher in patients without systemic TAA-specific T cell response, but these differences were also not significant. For stromal infiltration, patients with a systemic T cell response had a mean of 7.3 Tregs/HPF and patients without T cell response 12.2 Tregs/HPF (p = 0.11). The two patients in the limited stage of disease with a positive T cell response had a mean Treg epithelial infiltration of 2.0/HPF and a stromal infiltration of 8.7/HPF. The epithelial infiltration is similar to that of patients with limited disease and without T cell response; patients without T cell response in limited stages of disease had higher Treg stromal infiltration (14.1/HPF, significance not tested due to low patient number). The infiltration rates in metastatic disease – independent of stage – were similar to the two patients with limited disease and T cell response.

### Treg infiltration and survival in CRC

There was no difference in survival between patients with high total Treg infiltration and those with low total Treg infiltration, neither with nor without correction for the stage of CRC. In a descriptive post-hoc subset analysis a weakly better survival was found for patients with higher epithelial Treg infiltration after stage correction (p = 0.04).

### CD8 T cell infiltration in CRC and normal colon

CD8 infiltration was found in all CRC and healthy colon samples. Total CD8 T cell infiltration was significantly higher in CRC (28.5/HPF) than in healthy colon tissue (11.3/HPF, p = 0.03). While the CD8 infiltration of epithelial tissue was not significantly different between healthy colon (7.9/HPF) and CRC (9.5/HPF), stromal CD8 infiltration was significantly higher in CRC (19.0) than in the lamina propria of healthy colon (3.4/HPF, p < 0.01). Furthermore, in healthy tissue, epithelial CD8 T cell infiltration was significantly higher than infiltration of the lamina propria (p < 0.01). In contrast, in CRC, CD8 T cell infiltration was significantly higher in the stroma than in the epithelium (p < 0.01).

### CD8 T cell infiltration of CRC and Treg infiltration, stage and survival in CRC and

There was a weak positive correlation between Treg and CD8 (Pearson 0.625, p < 0.01) infiltration in the epithelium. No correlation was found between stromal Treg and CD8 infiltration (Pearson 0.273, n.s.). In all analyses, higher CD8 infiltration was associated with better survival. However, these data were not significant. There was only a statistical trend for better survival in patients with higher CD8 infiltration for stromal CD8 infiltration without stage correction (p = 0.06). There was a trend toward higher epithelial CD8 infiltration in the limited stage of CRC than in metastatic disease (p = 0.10). Stromal CD8 infiltration was increased in patients with limited CRC at a non-significant level (p = 0.33).

### Treg/CD8 ratio and survival in CRC; and age, gender and T cell infiltration of CRC

Since prior studies have analyzed ratios between Tregs and CD8 T cells in relation to survival, we also calculated the association of the Treg/CD8 ratio with survival. There was no association between higher or lower Treg/CD8 ratio and improved survival. No correlation was found between infiltration of tested T cell subpopulations (CD3, CD8, Treg) and age or gender.

## Discussion

Transcription factor forkhead box P3 (FOXP3) is a key intracellular marker and an important developmental and functional factor for CD4+ CD25+ regulatory T cells [39-41]. FOXP3 fulfills the criteria of a Treg-specific marker, which is, at least in differentiated Treg, not known to be substantially regulated and represents a good marker for quantifying Treg in tissues [31]. Here, we used FOXP3 staining in CRC to analyze Treg infiltration of CRC in situ and to test whether there is a correlation to a disease stage, systemic TAA-specific T cell response, and survival of CRC patients.

Tregs infiltrated all 40 CRC samples with higher Treg frequency in the stroma than in the tumor epithelium. In agreement with prior studies in various malignant diseases [16,26-28], we found significantly higher numbers of Tregs in CRC than in normal colon tissue. Although counts differ from patient to patient and, in particular, are relatively low in the epithelium, we found an approximately 20-fold increase of infiltrating Tregs in CRC compared to healthy colon.

Increased regulatory T cells in peripheral blood have been associated with advanced gastrointestinal tumor [15]. Interestingly, we found an approximately 2-fold increase of Tregs at the tumor site in CRC patients with limited compared to metastatic disease (p < 0.05). This difference is mainly based on a higher stromal Treg infiltration in limited CRC. The reason for this observation is unknown. However, it underlines the importance of *in situ *analyses. We speculate that migration of Tregs away from the primary tumor site during metastatic spread might be important in this setting, which would explain the Treg increase in peripheral blood as described by others. Furthermore, our data show that local Treg infiltration in malignant disease may be a dynamic process, with increased numbers as an initial response in limited disease followed by a relative decrease after metastatic spread.

In CRC, the average Treg infiltration rate was slightly higher in patients without systemic TAA-specific T cell response. Although these differences were not significant, this may be a first indication of a T cell response-suppressing role of tumor-infiltrating Tregs in CRC. In this context, the above described increased Treg infiltration in limited disease is particularly interesting because systemic T cell responses have been detected significantly less often in patients with limited disease (3, 4). This might suggest an indirect interaction between systemic T cell response and Treg infiltration. Of note, the herein presented T cell responses against the TAA EpCAM, her-2/neu, and CEA represent only a small proportion of potential T cell targets in CRC.

Evaluating a possible interaction between Treg infiltration and survival required a stage-corrected analysis due to the stage-dependency of survival and Treg infiltration. No association was found between total Treg infiltration and survival neither with nor without stage correction. In a descriptive post-hoc analysis a weakly better survival was found for patients with higher epithelial Treg infiltration after stage correction. This subset analysis must not be over-interpreted and must be seen with great caution because of multiple testing and the low number of epithelial Treg in CRC. Nonetheless, assuming that high Treg infiltration reduces anti-tumor immunity and thus survival, our results may seem surprising at first glance; and, e.g., in ovarian cancer, high Treg infiltration seems to be an adverse prognostic factor [29-31]. However, contrasting results are reported for various neoplasms. Erdman et al. [42] have shown that adoptive transfer of Tregs into mice significantly inhibited the development of microbially induced colon carcinoma. In a study on human Hodgkin lymphoma, it was shown that low FOXP3+ cell numbers correlated with negative clinical outcome [43]. Additionally, a recent study demonstrated that high numbers of tumor infiltrating FOXP3-positive regulatory T cells are associated with improved survival in follicular lymphoma [44]. It is possible that in some malignant disease – so far preferentially lymphoproliferative or bacteria-induced diseases – infiltrating Tregs may have a protective effect by reducing/delaying the development of an aggressive and cytotoxic, potentially proliferogenic cytokine milieu, which is the basis for the inflammation-driven progress of malignant diseases [45,46]. In accordance with our study, Pages et al. [47] measured FOXP3 at the RNA level in CRC and found no difference in survival comparing high and low expression.

Since CD8+ T cell tumor infiltration has been described as a positive prognostic factor in CRC [32-37], we performed an analysis of CD8 T cell infiltration in relation to disease stage and survival. First, based on our *in situ *approach, it was interesting to see that the increased CD8 infiltration in CRC compared to healthy colon was based on increased stromal CD8 tumor infiltration. Furthermore, we found a reversion of CD8 T cell distribution from higher epithelial CD8 infiltration in healthy colon to higher stromal CD8 infiltration in CRC suggesting a CD8 T cell shift to the stroma during tumor development. In accordance with the above mentioned previous studies, we found a trend for better survival in CRC patients with higher stromal CD8 infiltration. This may be due to an increase of CD8 infiltration in limited disease compared to metastatic disease as we found in our study. Thus, for further analyses on the influence of CD8 infiltration of CRC, it has to be taken into account that CD8 infiltration may be higher in the limited stages of the disease. Since ratios between Tregs and CD8 T cells in relation to survival were used in a prior study in ovarian cancer [30], we analyzed the association of the Treg/CD8 ratio with survival. No correlation was found. This difference between findings in ovarian cancer and CRC may be due to specific biological characteristics of each tumor type during progression with predominantly local spread in ovarian cancer and a higher tendency for systemic metastasis (e.g. to the liver) in CRC. While no correlation was found between stromal Tregs and CD8 infiltration in our study, it was interesting to see that Tregs and CD8 infiltration weakly correlated in the epithelial tissue.

Regulatory T cells are seen as a hindrance for clinically successful tumor-directed T cell responses particularly in discussions on active specific immunization and adoptive T cell transfer in cancer patients [25]. Our data do not directly support this assumption. There are only indirect links between T cell response and Treg infiltration: the Treg infiltration is higher in patients without systemic TAA-specific T cell response at a non-significant level, and Tregs are increased in patients with limited disease, in whom T cell responses are less frequent. We only begin to understand the relationships between tumor-infiltrating Treg, stage of disease, and systemic T cell response against TAA; and they have to be further investigated.

Our study adds new data to the ongoing discussion on the role of Tregs in malignant diseases. Our *in situ *analysis demonstrates a strong increase of Tregs in CRC compared to normal colonic mucosa and a predominant Treg presence in the stroma of CRC. It provides evidence that Treg infiltration is increased locally in limited stages of CRC. Furthermore, our data show that Treg infiltration is a rather dynamic process, in which Treg infiltration at the primary tumor site increases during early stages of CRC and decreases in advanced disease. Additionally, our study underlines the fact that each malignant disease seems to have its own characteristics concerning the influence of Treg infiltration on survival. Therefore, it suggests a precautionary approach during the modulation of the balanced system of Tregs and CD8 T cells by immuno- and chemotherapy.

## Abbreviations

CRC – colorectal cancer, Treg – regulatory T cell, FOXP3 – transcription factor forkhead box P3, HPF – high power field
